# Analysis of small and large subunit rDNA introns from several ectomycorrhizal fungi species

**DOI:** 10.1371/journal.pone.0245714

**Published:** 2021-03-15

**Authors:** Li-hong Chen, Wei Yan, Ting Wang, Yu Wang, Jian Liu, Zhuo Yu

**Affiliations:** 1 College of Horticulture and Plant Protection, Inner Mongolia Agricultural University, Huhhot, Inner Mongolia, China; 2 College of Forestry, Inner Mongolia Agricultural University, Huhhot, Inner Mongolia, China; 3 Ordos Institute of Technology, Ordos, Inner Mongolia, China; 4 College of Agronomy, Inner Mongolia Agricultural University, Huhhot, Inner Mongolia, China; Friedrich Schiller University, GERMANY

## Abstract

The small (18S) and large (28S) nuclear ribosomal DNA (rDNA) introns have been researched and sequenced in a variety of ectomycorrhizal fungal taxa in this study, it is found that both 18S and 28S rDNA would contain introns and display some degree variation in size, nucleotide sequences and insertion positions within the same fungi species (*Meliniomyces*). Under investigations among the tested isolates, 18S rDNA has four sites for intron insertions, 28S rDNA has two sites for intron insertions. Both 18S and 28S rDNA introns among the tested isolates belong to group I introns with a set of secondary structure elements designated P1-P10 helics and loops. We found a 12 nt nucleotide sequences TACCACAGGGAT at site 2 in the 3’-end of 28S rDNA, site 2 introns just insert the upstream or the downstream of the12 nt nucleotide sequences. Afters sequence analysis of all 18S and 28S rDNA introns from tested isolates, three high conserved regions around 30 nt nucleotides (conserved 1, conserved 2, conserved 3) and identical nucleotides can be found. Conserved 1, conserved 2 and conserved 3 regions have high GC content, GC percentage is almost more than 60%. From our results, it seems that the more convenient host sites, intron sequences and secondary structures, or isolates for 18S and 28S rDNA intron insertion and deletion, the more popular they are. No matter 18S rDNA introns or 18S rDNA introns among tested isolates, complementary base pairing at the splicing sites in P1-IGS-P10 tertiary helix around 5’-end introns and exons were weak.

## Introduction

Mycorrhizal symbiosis is a common phenomenon in all terrestrial plant communities. One of the major types of mycorrhiza is the ectomycorrhiza, typically formed by almost all tree species in temperate forests [1]. For the ectomycorrhiza symbiosis which the fungus forms a mantle external to the plant root, the number of plant and fungal species involved is currently estimated to be ca. 6,000 and 20,000–25,000, respectively [2, 3]. The ecologically and economically most important forest trees (Pinaceae, Fagaceae, Betulaceae, Nothofagaceae, Leptospermoideae of Myrtaceae, Dipterocarpaceae, and Amhersteae of Caesalpiniaceae, and so on) dominate woodland and forest communities in boreal, Mediterranean, and temperate forests of the Northern Hemisphere and parts of South America, seasonal savanna and rain forest habitats in Africa, India and Indo-Malay as well as temperate rain forest and seasonal woodland communities of Australia [4]. Mycorrhizal infection affects the mineral nutrition and micronutrient uptake of plants [5–7]. Based on taxonomic and ecological extrapolation, an estimated 86% of terrestrial plant species acquire mineral nutrients via mycorrhizal root symbionts [3]. For example, ectomycorrhizal fungus *Cenococcum graniforme* could produce ferricrocin, alkaline phosphatase and other hydrolyases to help hosts iron nutrient and carbohydrate utilization [1, 8]. Thus, ectomycorrhiza fungi play an important role in seedling establishment and tree growth in habitats across the globe.

Group I introns are small RNAs and are found in a wide variety of organisms (e.g. in fungi, algae and in many other unicellular eukaryotes), genes (i.e. protein, rRNA and tRNA coding genes) and genomes [9–11]. Group I introns spread effificiently at the DNA level into intronless cognate sites by homing process. Group I introns are characterized by the possession of a set of conversed sequences elements designated P1 and P3-P10. P4-P6 and P3-P9 helical domains constitute the catalytic core elements and P1 and P10 helical the substrate domain that contains the 5’ and 3’ splice sites [12–15]. Based on both conversed nucleotide sequences and secondary structure characterics, group I introns are classified into five major groups (IA to IE) according to the presence/absence of peripheral paired elements [14, 16].

In this study, the sequnences and deduced secondary structures of 18S and 28S rDNA introns have been examined among several fungal species. We would like to know the introns insertion positions in 18S and 28S rDNA, intron sequence homology, and their secondary structure features. We are also interested in compairing 18S rDNA introns with 28S rDNA introns in the respect of their similarities and differences, trying to find their evolution origin between 18S and 28S rDNA introns.

## Materials and methods

### Fungal strains and DNA extraction

Tested strains were isolated from sclerotial bodies as well as mycorrhizae samples which were collected from Daqing Mountain (longitude 111.25°-112.30°, Latitude 40.35°-40.57°) with permission from Inner Mongolia Daqing Mountain Nature Reserve, Helan Mountain (longitude 105.40°-105.58°, Latitude 38.10°-39.08°) with permission from Helan Mountain National Nature Reserve, Daxingan Mountainn (longitude 121.30°-121.31°, Latitude 50.49°-50.51°) with permission from Genhe ecological positioning station in Daxingan Mountainn of Inner Mongolia, and Wula Mountain (longitude 108.2°-108.5°, Latitude 40.9°- 40.41°) with permission from Inner Mongolia Wula Mountain National Forest Park in Inner Mongolia of China. No specific permits were required as the research did not include the destruction of vegetation. Information regarding the used isolates is provided in Table 1. For DNA extraction, mycelial plugs from stock cultures were grown on potato-dextrose agar (PDA) plates at 24°C for DNA extraction. Genomic DNA was extracted using a cetyltrimethyl ammonium bromide (CTAB) method [17], then stored at -20°C.

**Table 1:** Isolates used in this study.

Isolates	Host origin	Geographical origin
Spop1 (*Cenococcum geophilums*)	*Populus davidiana*	Daqing Mountain, China
Spop2 (*Cenococcum geophilums*)	*Populus davidiana*	Daqing Mountain, China
Spop3 (*Cenococcum geophilums*)	*Populus davidiana*	Daqing Mountain, China
Spop6 (*Cenococcum geophilums*)	*Populus davidiana*	Daqing Mountain, China
Spopx (*Cenococcum geophilums*)	*Populus davidiana*	Daqing Mountain, China
Pop4 (*Cenococcum geophilums*)	*Populus davidiana*	Daqing Mountain, China
Pop5 (*Cenococcum geophilums*)	*Populus davidiana*	Daqing Mountain, China
Pop2 (*Chaetothyriales*)	*Populus davidiana*	Daqing Mountain, China
Pop7 (*Chaetothyriales*)	*Populus davidiana*	Daqing Mountain, China
Yang1 (*Cenococcum geophilums*)	*Populus davidiana*	Daqing Mountain, China
SHY (*Cladophialophora*)	*Populus davidiana*	Daqing Mountain, China
O1 (*Cenococcum geophilums*)	*Ostryopsis daidiana*.	Daqing Mountain, China
O2 (*Cenococcum geophilums*)	*Ostryopsis daidiana*.	Daqing Mountain, China
O4 (*Cenococcum geophilums*)	*Ostryopsis daidiana*.	Daqing Mountain, China
O5 (*Cenococcum geophilums*)	*Ostryopsis daidiana*.	Daqing Mountain, China
SO1 (*Cenococcum geophilums*)	*Ostryopsis daidiana*.	Daqing Mountain, China
SO2 (*Pezizomycotina*)	*Ostryopsis daidiana*.	Daqing Mountain, China
SO4 (*Cenococcum geophilums*)	*Ostryopsis daidiana*.	Daqing Mountain, China
SO5 (*Cenococcum geophilums*)	*Ostryopsis daidiana*.	Daqing Mountain, China
Picea (*Meliniomyces*)	*Picea asperata*	Daqing Mountain, China
Spicea (*Cenococcum geophilums*)	*Picea asperata*	Daqing Mountain, China
B2 (*Cladophialophora*)	*Betula platypylla*	Daqing Mountain, China
B3 (*Cladophialophora*)	*Betula platypylla*	Daqing Mountain, China
B5(*Cladophialophora*)	*Betula platypylla*	Daqing Mountain, China
SB1 (*Cenococcum geophilums*)	*Betula platypylla*	Daqing Mountain, China
SB5 (*Cenococcum geophilums*)	*Betula platypylla*	Daqing Mountain, China
SB6 (*Pezizomycotina*)	*Betula platypylla*	Daqing Mountain, China
Quercus (*Cenococcum geophilums*)	*Quercus monogolica*	Daqing Mountain, China
MY (*Cenococcum geophilums*)	*Pinus tabulaeformis*	Daqing Mountain, China
Yang2 (*Meliniomyces*)	*Populus davidiana*	Daxingan Mountain, China
2010cg (*Cenococcum geophilums*)	*Betula platypylla*	Daxingan Mountain, China
Baihua (*Meliniomyces*)	*Betula platypylla*	Daxingan Mountain, China
Shanbai (*Meliniomyces*)	Unknown	Daxingan Mountain, China
WL (*Cenococcum geophilums*)	*Populus davidiana*	Wula Mountain, China
1–1 (*Cenococcum geophilums*)	*Picea asperata*	Helan Mountain, China
1–2 (*Cenococcum geophilums*)	*Picea asperata*	Helan Mountain, China
1–3 (*Cenococcum geophilums*)	*Picea asperata*	Helan Mountain, China
YUN (*Cenococcum geophilums*)	*Picea asperata*	Helan Mountain, China
2–1 (*Cenococcum geophilums*)	*Populus davidiana*	Helan Mountain, China
2–2 (*Cenococcum geophilums*)	*Populus davidiana*	Helan Mountain, China
2–3 (*Cenococcum geophilums*)	*Populus davidiana*	Helan Mountain, China
2–4 (*Cenococcum geophilums*)	*Populus davidiana*	Helan Mountain, China
2–5 (*Cenococcum geophilums*)	*Populus davidiana*	Helan Mountain, China
2–6 (*Cenococcum geophilums*)	*Populus davidiana*	Helan Mountain, China
2–7 (*Cenococcum geophilums*)	*Populus davidiana*	Helan Mountain, China
2–8 (*Cenococcum geophilums*)	*Populus davidiana*	Helan Mountain, China
2–9 (*Cenococcum geophilums*)	*Populus davidiana*	Helan Mountain, China
2–10 (*Cenococcum geophilums*)	*Populus davidiana*	Helan Mountain, China
2–11 (*Cenococcum geophilums*)	*Populus davidiana*	Helan Mountain, China
2–12 (*Cenococcum geophilums*)	*Populus davidiana*	Helan Mountain, China
2–13 (*Cenococcum geophilums*)	*Populus davidiana*	Helan Mountain, China
2–14 (*Cenococcum geophilums*)	*Populus davidiana*	Helan Mountain, China
2–15 (*Pezizomycotina*)	*Populus davidiana*	Helan Mountain, China
2–16 (*Chaetothyriales*)	*Populus davidiana*	Helan Mountain, China
2–17 (*Phialophore verrucosa*)	*Populus davidiana*	Helan Mountain, China
3–1 (*Cenococcum geophilums*)	*Pinus tabulaeformis*	Helan Mountain, China
3–2 (*Cenococcum geophilums*)	*Pinus tabulaeformis*	Helan Mountain, China
3–3 (*Cenococcum geophilums*)	*Pinus tabulaeformis*	Helan Mountain, China
3–4 (*Cenococcum geophilums*)	*Pinus tabulaeformis*	Helan Mountain, China
4–1 (*Cenococcum geophilums*)	*Jumiperus communis*	Helan Mountain, China
CG (*Cenococcum geophilums*)	Unknown	France
CG5 (*Cenococcum geophilums*)	Unknown	France
CG54 (*Cenococcum geophilums*)	Unknown	France
CG417 (*Cenococcum geophilums*)	Unknown	France
AM51 (*Meliniomyces*)	Unknown	France
CGTAR (*Cenococcum geophilums*)	Unknown	Switzerland
CGPIL (*Cenococcum geophilums*)	Unknown	Switzerland
CGLESPAC (*Cenococcum geophilums*)	Unknown	Switzerland
010 (*Cenococcum geophilums*)	*Pinus resinosa Ait*.	USA
011 (*Cenococcum geophilums*)	*Pinus resinosa Ait*.	USA
155 (*Cenococcum geophilums*)	*Quercus alba L*.	USA
ALB-2 (*Cenococcum geophilums*)	*Abies lasiocarpa* Nutt.	USA
S8-1 (*Cenococcum geophilums*)	*Picea glauca* Vess.	USA
HUNT-8 (*Cenococcum geophilums*)	*Picea rubrens* Sargent	USA
HUNT-9 (*Cenococcum geophilums*)	*Picea rubrens* Sargent	USA
1-1-4 (*Cenococcum geophilums*)	*Quercus douglasii*	USA
1-7-7 (*Cenococcum geophilums*)	*Quercus douglasii*	USA
1-7-8 (*Cenococcum geophilums*)	*Quercus douglasii*	USA
1-7-11 (*Cenococcum geophilums*)	*Quercus douglasii*	USA
1-19-1 (*Cenococcum geophilums*)	*Quercus douglasii*	USA
2-3-1 (*Cenococcum geophilums*)	*Quercus douglasii*	USA
2-6-1 (*Cenococcum geophilums*)	*Quercus douglasii*	USA
2-10-3 (*Cenococcum geophilums*)	*Quercus douglasii*	USA
2-11-1 (*Cenococcum geophilums*)	*Quercus douglasii*	USA
2-13-2 (*Cenococcum geophilums*)	*Quercus douglasii*	USA
2-14-4 (*Cenococcum geophilums*)	*Quercus douglasii*	USA
3-2-5 (*Cenococcum geophilums*)	*Quercus douglasii*	USA
3-7-3 (*Cenococcum geophilums*)	*Quercus douglasii*	USA
3-9-2 (*Cenococcum geophilums*)	*Quercus douglasii*	USA
3-10-2 (*Cenococcum geophilums*)	*Quercus douglasii*	USA
3-10-3 (*Cenococcum geophilums*)	*Quercus douglasii*	USA
3-11-1 (*Cenococcum geophilums*)	*Quercus douglasii*	USA
3-18-1 (*Cenococcum geophilums*)	*Quercus douglasii*	USA
1-5-4 (*Cenococcum geophilums*)	*Quercus douglasii*	USA
3-4-II (*Cenococcum geophilums*)	*Quercus douglasii*	USA
I-2 (*Cenococcum geophilums*)	*Quercus douglasii*	USA
I-3 (*Cenococcum geophilums*)	*Quercus douglasii*	USA
BTREE1 (*Cenococcum geophilums*)	*Quercus douglasii*	USA

### PCR amplification and sequencing

The 3’-end of 18S rDNA was amplified using primers NS5 (5’-GATACCGTCGTATCTTAACC-3’) / NS8 (5’-TCCGCAGGTTCACCTACGGA-3’) [15]. An initial denaturation at 94°C for 5min was followed by 30 cycles of denaturation at 94°C for 30s, annealing at 50°C for 30s, and extension at 72°C for 90s. There was a final extension step at 72°C for 10min. The 3’-end of 28S rDNA was amplified using primers Vdahl4 (5’-CGGGCTTGGCAGAATCAG-3’) / Vdahl2 (5’-GCGACGTCGCTATGAACG-3’) [18]. An initial denaturation at 94°C for 1min was followed by 30 cycles of denaturation at 94°C for 30s, annealing at 47°C for 30s, and extension at 72°C for 90s. There was a final extension step at 72°C for 10min. 18S rDNA-ITS-28S rDNA region was amplified using primers ITS1 (5′-TCCGTAGGTGAACCTGCGG-3′) / ITS4 (5′-TCCT CCGCTTATTGATATGC-3′) [19]. An initial denaturation at 94°C for 1min was followed by 30 cycles of denaturation at 94°C for 30s, annealing at 50°C for 30s, and extension at 72°C for 120s. There was a final extension step at 72°C for 10min. The products were electrophoresed in a 1% (w/v) agarose gel to check the efficiency of amplification. The purified amplicons were sequenced by Shanghai Sangon Biotechnology Co., Ltd, Shanghai Invitrogen Biotechnology Co., Ltd, Beijing Tsingke Biotechnology Co., Ltd., China. The sequences were aligned by sequence analysis software DNAMAN, Lynnon Corporation.

### Intron secondary structure modeling

Secondary structure models were predicted following the conventions for group I introns defined by Burke et al. and according to the models proposed by Cech and Michel and Westhof [12–14]. The P1-P9 stem-loop elements were individually identified by comparison with available group I intron sequences from the Comparative RNA web site (CRW at http://www.rna.icmb.utexas.edu/) and then folded using the mfold web server at http://www.bioinfo.rpi.edu/applications/mfold/old/rna/form1.cgi [20, 21]. The RNA secondary structures were calculated and drawn using RNAstructure version 4.6 [22].

## Results

### Positions and structure analysis of 18S rDNA introns

**Fig 1:**
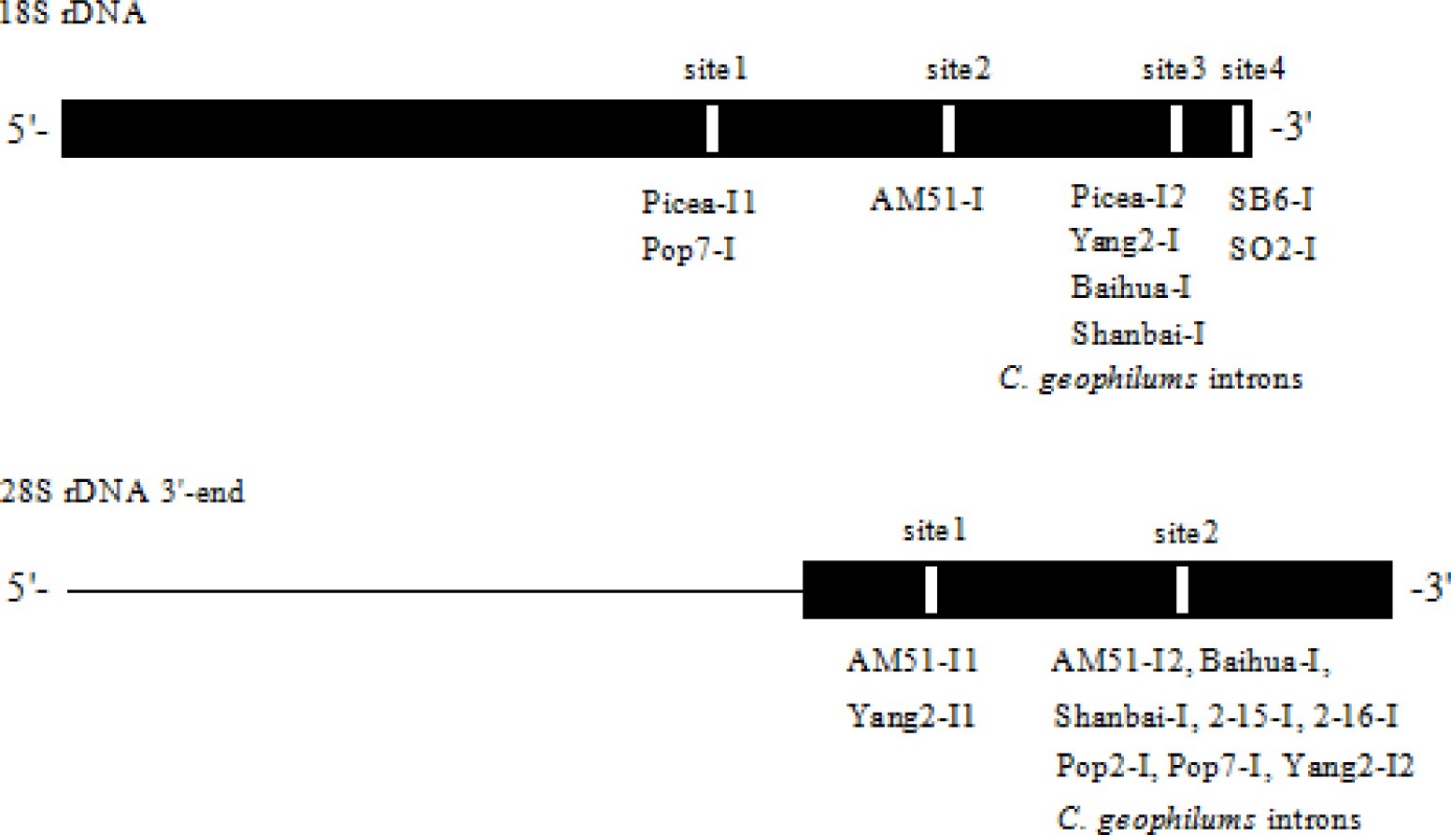
The positions of intron insertion in 18S and 28S rDNA of tested isolates.

**Fig 2:**
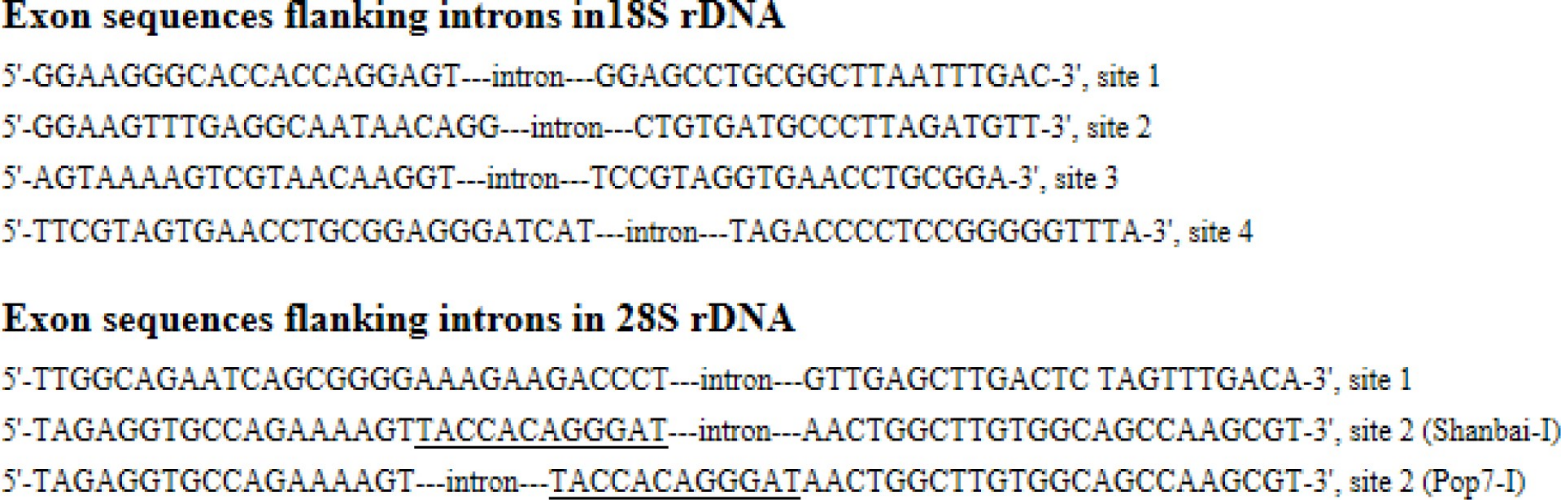
The exon sequences flanking introns in 18S and 28S rDNA of tested isolates. Exon sequences flanking introns in 28S rDNA, site 1, 5’-end sequences from *Pezizomycotina* 28S rDNA in GenBank, 3’-end sequences from isolate AM51 this study.

The 18S rDNA 3’-end of tested isolates (AM51, Baihua, Shanbai, Picea, Yang2, Pop7, SB6, SO2, B2, B3, B5, 2–15, 2–16, 2–17, SHY) was PCR amplified by primers NS5 / NS8, 18S rDNA-ITS-28S rDNA region isolates (SB6, SO2) was PCR amplified by primers ITS1 / ITS4, and then sequenced. After sequencing it was found that the isolates AM51, Baihua, Picea, Shanbai, Yang2, Pop7, SO2, SB6 possessed the introns, while the isolates 2–15, 2–16, 2–17, B2, B3, B5, SHY did not contain introns in 18S rDNA 3’-end. We found 18S rDNA of the tested isolates has four sites for intron insertions, the introns (Picea-I1, Pop7-I) insert at the same site in 18S rDNA sequence (site 1), the intron (AM51-I) insert at site 2, the introns (Picea-I2, Yang2-I, Baihua-I, Shanbai-I, Spop1-I, O5-I) insert at site 3, the introns (SB6-I, SO2-I) insert at site 4. Isolate Picea has two different type introns (Picea-I1 and Picea-I2) at the 3’-end of 18S rDNA, distributing at site 1 and site 3. The 18S rDNA full length of isolates Picea, Shanbai, AM51, Spop1, O5, CG54 were sequenced, there was no introns found at the 5’-end of 18S rDNA. The intron distribution in 18S rDNA of tested isolates in this study was showed in Fig 1, the exon sequences flanking introns were showed in Fig 2.

**Fig 3:**
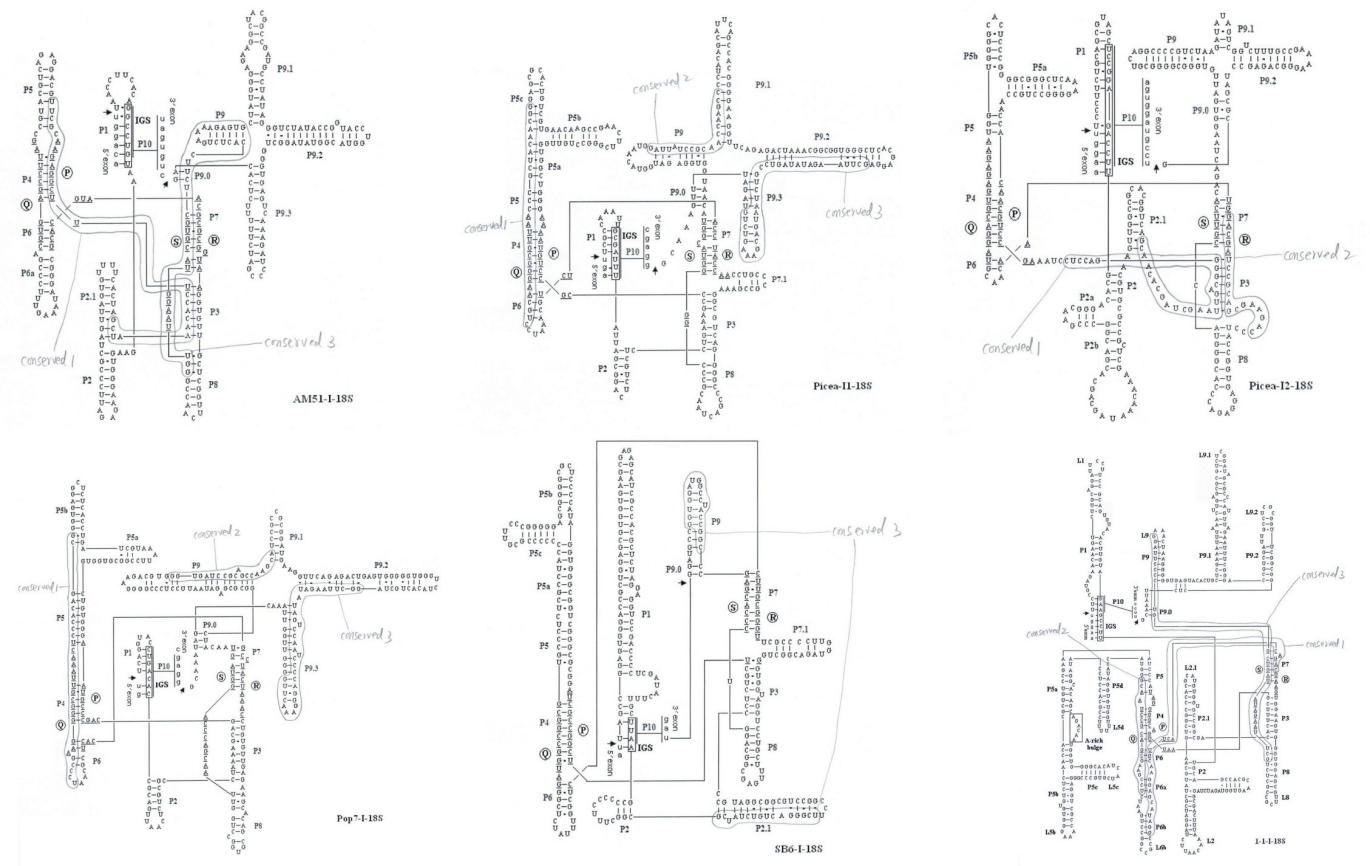
Secondary structures of 18S rDNA introns AM51-I, SB6-I, Pop7-I, Picea-I1, Picea-I2, 1-1-I. The nucleotides of the 18S rDNA intron are indicated in capital letters, while the flanking exons are in lower case letters. Arrows denote the 5’ and 3’ splice sites. Nucleotides within the conserved core element P, Q, R and S regions are underlined. The IGS region and A-rich bulge are indicted by boxes surrounding the sequences. Conerved 1, conserved 2, and conserved 3 regions are rounded by light line.

Fig 3 showed that the deduced secondary structures of 18S rDNA introns (AM51-I, SB6-I, Pop7-I, Picea-I1, Picea-I2, 1-1-I) from tested isolates had the same features known to be conserved among group-I introns: the last exon base U and the last intron base G; the pairing regions P1-P10; the consensus elements P, Q, R and S within the core region; the internal guide sequences (IGS) proposed to help align the exons for splicing [23–29]. Beside these common structures of group-I introns above, the 18S rDNA introns (Picea-I1, Picea-I2, Pop7-I, SB6-I, 1-1-I, Spicea-I) have an extensive P5 region (P5, P5a, P5b, P5c and P5d), the 18S rDNA introns (Picea-I1, Picea-I2, Pop-I, AM51-I, 1-1-I, Spicea-I) have two extra stems on the 3’ side of P9 (P9.1 and P9.2) from this study and we reported previously [30, 31]. The 18S rDNA intron (Picea-I1, Picea-I2, Pop7-I, AM51-I, 1-1-I, Spicea-I) possess an A-rich bulge, however, we did not find an typical A-rich bulge around P5 pairing region in the secondary structures of 18S rDNA intron SB6-I. The sequences of Picea-I2, Yang2-I, Baihua-I, Shanbai-I exhibited 94.7% identity, they have the same secondary structure. The sequences of SB6-I and SO2-I exhibited 98.8% identity, they have the same secondary structure. Picea-I1 and Pop-I have quite low sequence identity (61%), but still have quite similar secondary structures.

### Positions and structure analysis of 28S rDNA introns

The 28S rDNA 3’-end of tested isolates (Spop1, Spop2, Spop3, Pop4, Pop5, Spop6, Spopx, Pop2, Pop7, O1, O2, O4, O5, SO1, SO2, SO4, SO5, SB1, SB2, SB5, SB6, 1–2, 2–1, 2–2, 2–4, 2–5, 2–6, 2–7, 2–8, 2–9, 2–10, 2–12, 2–14, 2–15, 2–16, 2–17, 3–1, 3–3, 3–4, 4–1, WL, 2010cg, MY, AM51, Baihua, Shanbai, Yang2, B2, B3, B5, CG5, CG417, CG54) was amplified by PCR and sequenced. After sequencing it was found that the isolates Spop1, Spop3, Pop4, Spop6, Spopx, O1, O2, O4, O5, SO1, SO5, SB1, SB5, 2–2, 2–5, 2–6, 2–7, 2–8, 2–12, 2–15, 2–16, 3–1, 3–4, WL, 2010cg, CG5, CG417, CG54, AM51, Yang2, Baihua, Shanbai, Pop2, Pop7 possessed introns, the isolates Pop5, Spop2, MY, SO2, SO4, SB2, SB6, 1–2, 2–1, 2–4, 2–9, 2–10, 2–14, 2–17, 3–3, 4–1, B2, B3, B5 did not have introns. 28S rDNA 3’-end has two sites for intron insertions (Fig 1). Except isolates AM51 and Yang2 have two types introns (AM51-I1, AM51-I2, Yang2-I1, Yang2-I2) and insert at site 1 and site 2, the other introns (Shanbai-I, Baihua-I, Pop2-I, Pop7-I, 2-15-I, 2-16-I, and all *Cenococcum geophilums* introns) insert at site 2. We found a 12 nt nucleotide sequences TACCACAGGGAT at site 2 in the 3’-end of 28S rDNA. Introns AM51-I2, Baihua-I, Picea-I, Shanbai-I, 2-15-I, 2-16-I, and all tested *Cenococcum geophilums* introns just insert in the downstream of the12 nt nucleotide sequences, while introns Pop2-I, Pop7-I just insert in the upstream of the 12 nt nucleotide sequences (Fig 2). The intron distribution in 28S rDNA of tested isolates in this study was showed in Fig 1, the exon sequences flanking introns were showed in Fig 2. Intron distribution compairson between18S rDNA and 28S rDNA were listed in Table 2. Some isolates have both 18S and 28S rDNA introns, some isolates have one of 18S or 28S rDNA introns, some isolates have neither 18S or 28S rDNA introns. Among tested isolates, AM51, Picea, Yang2, Shanbai, Baihua, belong to *Meliniomyces* spesice, both 18S and 28S rDNA introns display some degree variation in size, nucleotide sequences and insertion positions. While all tested *Cenococcum geophilums* 18S introns insert at site 3 and 28S introns insert at site 2, both sequences display high homology, respectively.

**Table 2:** Intron distribution patterns of 18S and 28S rDNA in tested isolates.

Isolate	18S Intron	28S Intron	Isolate	18S Intron	28S Intron	Isolate	18S Intron	28S Intron	Isolate	18S Intron	28S Intron
O1	−	+	Pop5	+	+	YUN	+	+	2–13	+	+
O2	−	+	Yang1	+	UN	2–1	−	−	2–14	−	−
O4	+	+	Quercus	−	UN	2–2	−	+	3–1	+	+
O5	+	+	2010cg	+	+	2–3	−	+	3–2	+	UN
SO1	+	+	SB1	+	+	2–4	+	−	3–3	+	−
SO4	−	−	SB2	−	−	2–5	−	+	3–4	−	+
SO5	−	+	SB5	+	+	2–6	−	+	4–1	+	−
Spop1	+	+	Spicea	+	+	2–7	−	+	CG5	+	+
Spop2	+	−	MY	+	−	2–8	−	+	CG417	+	+
Spop3	+	+	WL	−	+	2–9	+	−	CG5	+	+
Spop6	+	+	1–1	+	UN	2–10	−	−	CG	+	UN
Spopx	+	+	1–2	+	−	2–11	−	+	2–15	−	+
Pop4	+	−	1–3	+	UN	2–12	−	+	2–16	−	+
2–17	−	−	Picea	+	UN	SB6	+	−	SO2	+	−
AM51	+	+	Pop7	+	+	Pop2	UN	+	Yang2	+	+
Shanbai	+	+	Baihua	+	+	B3	−	−	B5	−	−
SHY	−	UN	B2	−	−						

“+”: presence of intron; “−”: absence of intron; “UN”: unknown

**Fig 4:**
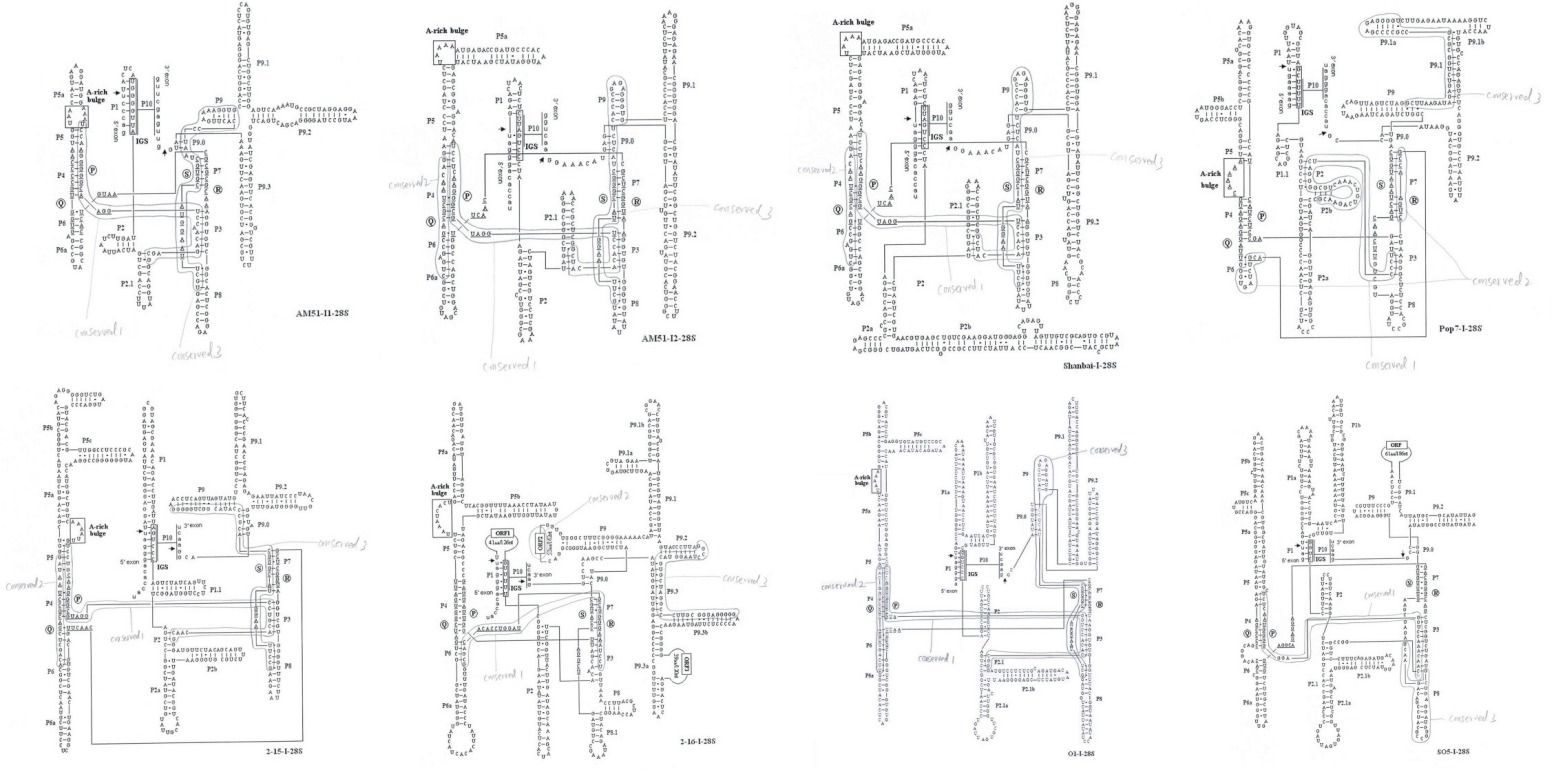
Secondary structures of 28S rDNA introns AM51-I1, AM51-I2, Shanbai-I, Pop7-I, 2-15-I, 2-16-I, O1-I, O1-I, SO5-I. The nucleotides of the 18S rDNA intron are indicated in capital letters, while the flanking exons are in lower case letters. Arrows denote the 5’ and 3’ splice sites. Nucleotides within the conserved core element P, Q, R and S regions are underlined. The IGS region and A-rich bulge are indicted by boxes surrounding the sequences. Conerved 1, conserved 2, and conserved 3 regions are rounded by light line.

Fig 4 showed that the deduced secondary structures of 28S rDNA introns (AM51-I1, AM51-I2, Shanbai-I, Pop7-I, 2-15-I, 2-16-I, O1-I, SO5-I) from the tested isolates had the same features known to be conserved among group-I introns: the last exon base U and the last intron base G; the pairing regions P1-P10; the consensus elements P, Q, R and S within the core region; the internal guide sequences (IGS) necessary for alignment of the two exons for splicing; the same insertion positions (site 2) compaired with other group-I introns. Beside these common structures of group-I introns above, all tested 28S rDNA introns have an A-rich bulge around P5 pairing region, an more or less extensive P5 region, and extra stems on the 3’ side of P9 (P9.1, P9.2, P9.3).

**Fig 5:**
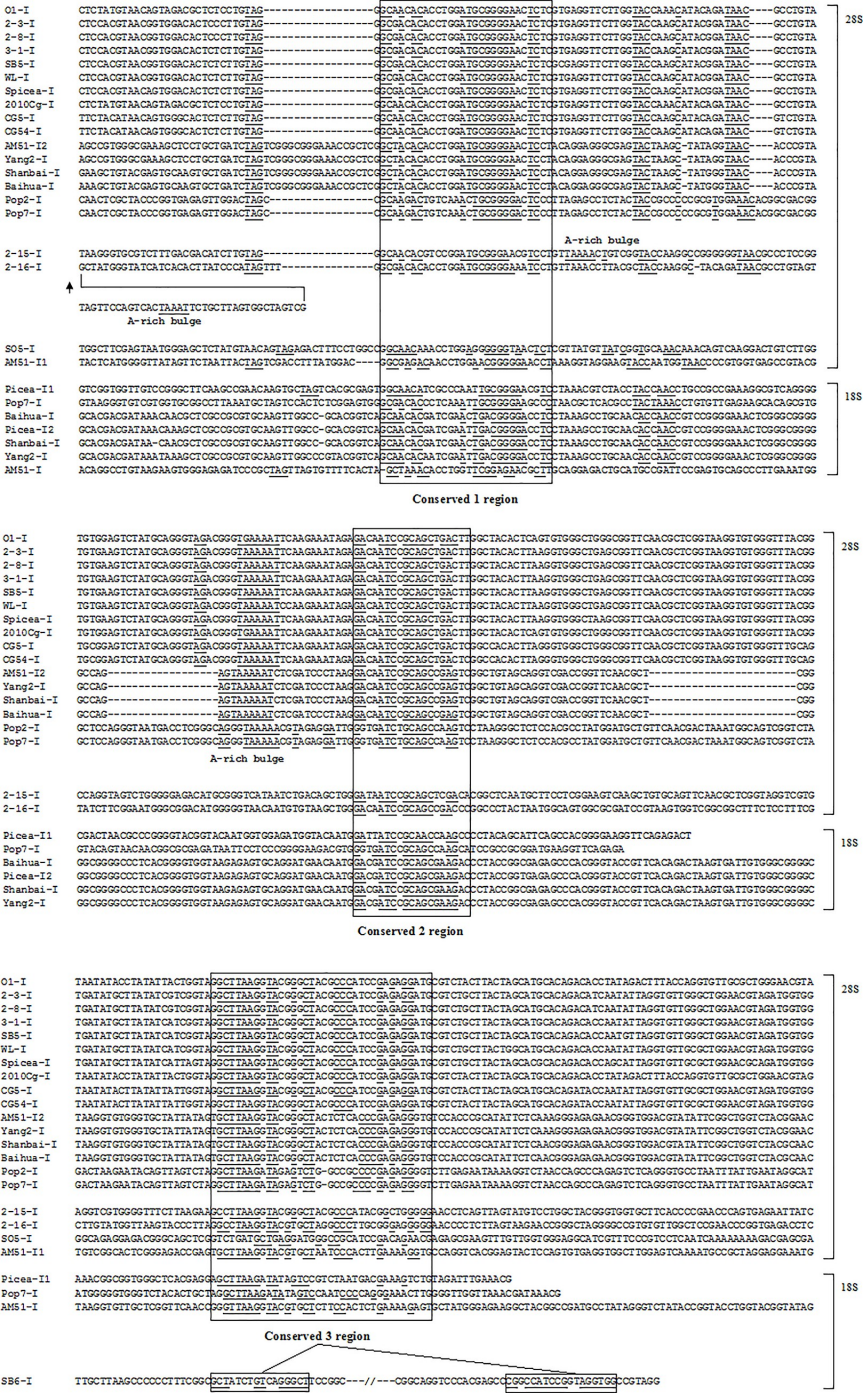
Positions of conserved 1, conserved 2, and conserved 3 regions in 28S and 18S rDNA introns. Top column of intron sequences are continuous from beginning to end. Below columns of intron sequences are extracted corresponding sequences. Identical nucleotides are underlined. Conserved 1, conserved 2, and conserved 3 regions are originally found in 28S rDNA introns.

Sequence analysis of 28S rDNA site 2 introns (AM51-I2, Yang2-I2, Picea-I, Shanbai-I, Baihua-I, Pop2-I, Pop7-I, 2-15-I, 2-16-I, and all *Cenococcum geophilums* introns) from tested isolates, it was found three high conserved regions around 30 nt nucleotides (conserved 1, conserved 2, conserved 3), and identical nucleotides can be found in the three conserved regions (Fig 5). Conserved 1, conserved 2 and conserved 3 regions have high GC content, GC percentage is almost more than 60%, that implied conserved 1, conserved 2, conserved 3 regions take part in complementary base pairing which maybe more firm. Sequence analysis of the three high conserved regions combining with deduced intron RNA secondary structures, three high conserved regions maybe participate in forming P3, P7, P4, helices- core region (the consensus elements P, Q, R and S within the core region), or important for maintaining core region structure, or splicing founction. Conserved 1 region distributes around P3 and P4 helices, and can pull P3 and P4 helices together. Conserved 2 region distributes around P4, P6, P7 helices, that maybe make P Q consensus elements in P4 helix more stable (conserved 2 region can pair with conserved 1 region in many introns, for example AM51-I2, Shanbai-I, 2-15-I, and all tested *Cenococcum geophilums* introns.), or can pull P6 and P7 helices together (conserved 2 region distributes around P6 and P7 helices in introns Pop7-I and Pop2-I). Conserved 2 region in intron 2-16-I can be found in P9 helix unpairing region, in which small ORF can be found. Conserved 2 region did not be found in intron SO5-I. Conserved 3 region distributes around P7, P8, P9, maybe important for strengthening core region secondary structure, or important for forming loop L8, L9, L9.1, L9.2, L9.3 (Fig 4). According to their distributions in introns, there are three conditions: (1) Conserved 1, conserved 2, and conserved 3 regions all maybe pull the consensus elements P, Q, R and S together to make the core region of secondary structure more stable and form loop L9 in tested introns AM51-I2, Yang2-I2, Picea-I, 2-15-I, Shanbai-I, Baihua-I, and all *Cenococcum geophilums* introns; (2) Conserved 1 and conserved 2 regions maybe pull the elements P, Q, R and S together, or make the core region more stable in tested introns Pop2-I, Pop7-I, conserved 3 region maybe important for P9 helice to form loop L9.1a; (3) Introns 2-16-I and SO5-I, only conserved 1 maybe pull the elements P, Q, R and S together, conserved 2 and conserved 3 maybe important for P9 helix to form loop L9 and L9.3. (1) type has majority tested introns, (1) type introns maybe more stable, suitable or highly efficient for intron insertion and deletion. Comparing tested intron sequences, conserved 1 region is more conservative than conserved 2 and conserved 3 regions. Conserved 3 region seems more conservative than conserved 2 region. Conserved 1 region seems more important for intron core region structure maintaining. Conserved 1, conserved 2 and conserved 3 regions in introns 2-16-I and SO5-I, containing long unpairing nucleotide sequence with small HEG ORFs, overall are less conservative than introns without HEG ORFs. The introns containing HEGs can be spliced by homing endonucleases, and endonuclease-mediated intron homing is an effificient process. Homing is initiated by an intron-encoded homing endonuclease that recognizes and generates a double-stranded DNA break close to the site of intron insertion [32–40]. Because introns containing HEGs can code themself endonucleases to splice introns, probably they did not need conserved sequences too much, or dependent on conserved sequences completely. This maybe the reason why sequences of introns containing HEGs are less conservative than introns without HEGs.

Sequence analysis of 28S rDNA site 1 introns (AM51-I1 and Yang2-I1) from isolates AM51 and Yang2, conserved 1 and conserved 3 regions still can be found. Sequence analysis of conserved 1, 3 regions combining with intron secondary structures, conserved 1 region distributes around P3 and P4 helices and can pull them together, conserved 3 region distributes around P7, P8, P9, maybe important for strengthening core region secondary structure, or important for forming loop L9 (Fig 5). Conserved 2 region did not find in introns AM51-I1 and Yang2-I1.

**Fig 6:**
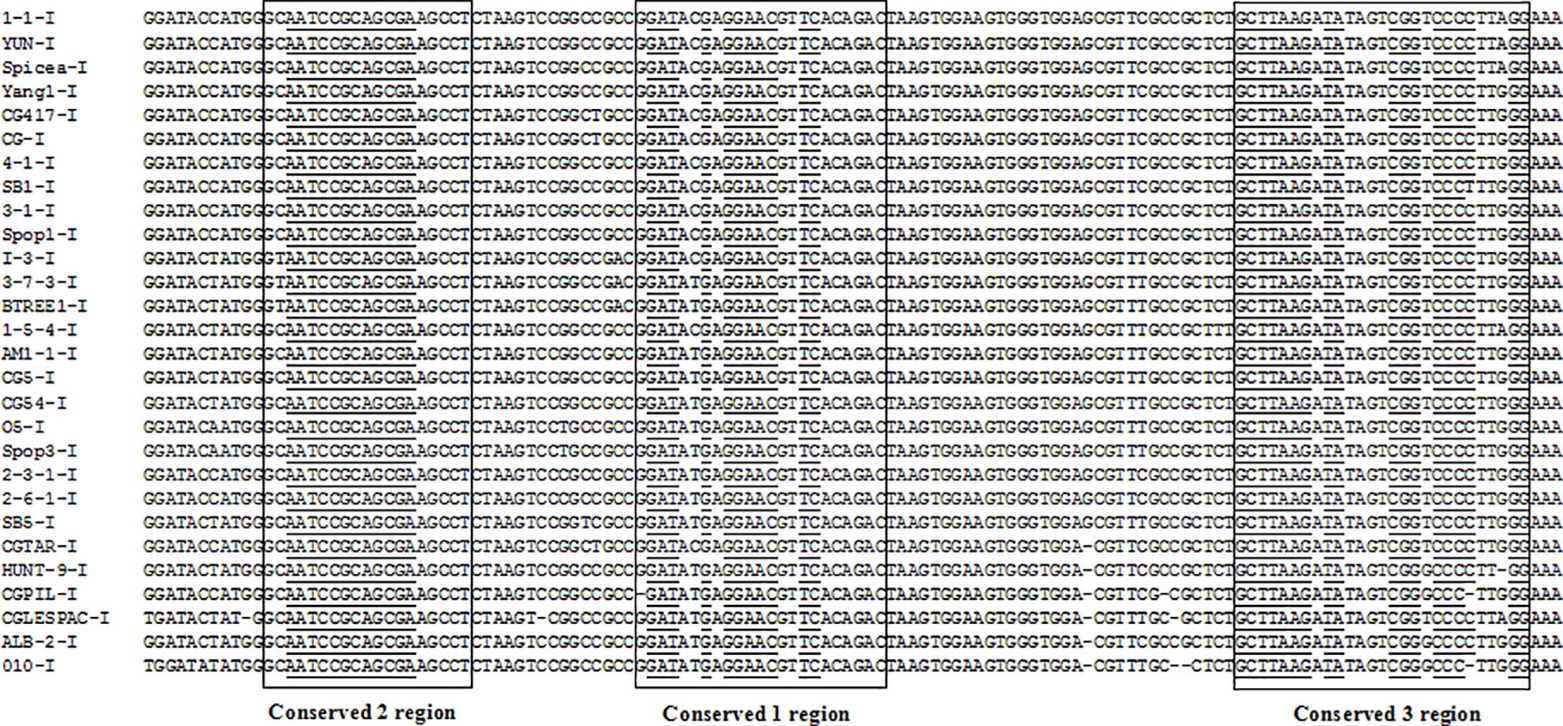
Positions of conserved 1, conserved 2, and conserved 3 regions in *Cenococcum geophilums* 18S rDNA introns. Intron sequences are continuous from beginning to end. Identical nucleotides are underlined. Conserved 1, conserved 2, and conserved 3 regions are originally found in 28S rDNA introns.

We would try to find out whether the 28S intron conserved 1, 2, 3 regions exist in 18S rDNA introns or not, interestingly the trace of 28S intron conserved 1, 2, 3 regions can be found in 18S rDNA introns (Figs 5 and 6). Conserved 1, conserved 2 and conserved 3 can be found in all *Cenococcum geophilums* 18S rDNA introns listed in Table 1 (site 3), differently just conserved 2 located in the upstream of conserved 1, but conserved 2 still can pair with conserved 1 (Fig 6). *Cenococcum geophilums* is an ecologically important ectomycorrhizal fungus with a global distribution and a broad host range [41], if there is a reason because its 18S and 28S rDNA intron sequences and secondary structures are easy for insertion and deletion? Conserved 1, conserved 2 and conserved 3 can be found in 18S rDNA introns Picea-I1 and Pop7-I (site 1). Conserved 1 and conserved 2 can be found in 18S rDNA introns Picea-I2, Yang2-I, Baihua-I, Shanbai-I (site 3). Conserved 1 and conserved 3 can be found in 18S rDNA intron AM51-I (site 2). Only conserved 3 can be found in 18S rDNA intron SB6-I (site 4), but was divided into two part, 5’-end located in P2.1 helix, 3’-end located in helix P9 and loop L9 (Fig 3).

## Discussion

Intron 2-16-I and SO5-I, beside pairing regions P1-P10, they have long unpairing regions, try to find open reading frame and seem they contain small ORFs, maybe they belong to HEG-associated group I introns (Fig 4). Goddard and Burt (1999) published a model of intron life-cycle and homing that involved intron cyclical gain and loss. Full-length HEG maybe need for invading, once the intron becomes fixed, the HEG no longer need, therefore it will accumulate mutations and become non-founctional or lost HEG [42]. From this evoluation point of view, the introns without HEG genes maybe advanced, the introns containing HEG genes maybe old. We found conserved 1, 2, 3 regions from introns 2-16-I and SO5-I with HEG are less conservative than as the introns without HEG did. Introns containing HEG are very rare among 18S rDNA and 28S rDNA, we only found three introns containing HEG (SB5-I from 18S rDNA, SO5-I and 2-16-I from 28S rDNA) from our all tested 18S rDNA and 28S rDNA sequences. The HEG gene no longer need, will be gradually deleted, 2-16-I and SO5-I seem have residual HEG gene nucleotides (non-founctional nucleotide sequences). The reason why residual HEG gene (non-founctional nucleotide sequences) still remain in intron sequences, probably because residual HEG genes have nucleotides which take part in intron secondary structure maintaining or founctions. We did not find the introns containing full length HEG gens, three introns containing HEG (SB5-I from 18S rDNA, SO5-I and 2-16-I from 28S rDNA) all contain residual HEG genes about 100–200 nucleotide sequences, from our isolated ectomycorrhizal fungal samples, our sample all were collected China.

The 12 nt nucleotide sequences TACCACAGGGAT at site 2 in the 3’-end of 28S rDNA, which is just upstream or downstream of the intron insertion position, the high conserved regions and identical nucleotide sequences in the introns at site 2, maybe much easier for introns to insert or delete. Introns break the integrality of exons sequences, introns possibly could control exon genes expressing. we can find 18S rDNA and 28S rDNA absence and presence of introns in the same isolate, for example, isolate CG5 has both 18S rDNA absence and presence of introns. We also find other isolates have both 18S rDNA absence and presence of introns. Genome DNA contains many 18S-5.8S-28S rDNA repeat unit, if product protein expressing from 28S rDNA is over-expressed more than cell metabolization need, will accumlate in cell. Product protein expressing from 28S rDNA is larger than from 18S rDNA, over-expression of 28S rDNA probably increase the cells more burden than over-expression of 18S rDNA. So the mechanism of 28S rDNA expressing control maybe more convenient than 18S rDNA expressing control, intron maybe one of the gene expressing controls. The majority of isolates contain 18S and 28S rDNA introns from our population genetic structure analysis previously, which means isolates containing 18S and 28S rDNA introns are more popular than isolates without 18S and 28S rDNA introns, furthermore, which imply that isolates containing 18S and 28S rDNA introns fit selection pressure better than isolates without 18S and 28S rDNA introns. Probably, the population genetic structure with absence and presence of 18S and 28S rDNA introns are in the balance of gain and lost 18S and 28S rDNA introns. The presence rate of *Cenococcum geophilums* 18S rDNA introns from China, America, Europe is significantly different from reports and our work, maybe the presence rate of 18S rDNA introns fit the selection pressure coming from its geographical origin. Europe temperature overall is colder than China, whether the presence rate of introns and evolution speed of plant host and fungus are affected by temperature?

Weeks and Cech reported that the yeast mitochondrial group I intron b15 undergoes self-splicing at high Mg^2+^ concentrations, but requires the splicing factor CBP2 for reaction under physiological conditions. Protein CBP2 could help assembly of the catalytic core, which involves association of two domains with each other and with other peripheral structures, and help association of the 5’ domain containing the 5’ splice site with the catalytic core properly [43]. The *Tetrahymena* preribosomal RNA intron could undergoes self-splicing in the absence of any proteins [44, 45]. Analysis the P1-IGS-P10 tertiary helix between 5’-end introns and exons in 18S and 28S rDNA in this study, we found that the complementary base pairing around the splicing sites were weak. In the P1-IGS-P10 tertiary helix around the splicing sites, there are many UG base pairing and unpairing bases. One of the group-I intron features known to be conserved is the last exon base U. UA and UG bonds are weaker than CG bond, and the presence of unpairing bases could also make the complementary base pairing helix unstable in same degree. The 5’ and 3’ exons both base pair to the intron’s IGS resulting in P1 and P10 helix formation, respectively [45], UG base pairing and unpairing bases in P1-IGS-P10 tertiary helix between 5’-end introns and exons maybe make introns easy to be cut off and make 5’ and 3’ exons easy to be ligation. Other papers indicated that 5’ splice site in P1-IGS-P10 tertiary helix possess UG bond quite common, in almost all introns present a UG pair at the 5’ splice site [24, 46–49].

From the results above, introns in 28S rDNA are much easier to find conserved 1, 2, 3 region than introns in 18S rDNA; site 3 in 18S rDNA introns and site 2 in 28S rDNA introns are hot positions for intron insertion, introns located at site 3 in 18S rDNA and site 2 in 28S rDNA are much easier to find conserved 1, 2, 3 regions than site 1, 2, 4 in 18S rDNA introns and site 1 in 28S rDNA introns; *Cenococcum geophilums* is one of the most popular ectomycorrhizal fungi, introns in both 18S rDNA and 28S rDNA are much easier to find conserved 1, 2, 3 regions than other fungal species. It seems that the more convenient host sites, intron sequences and secondary structures, or isolates for 18S and 28S rDNA intron insertion and deletion, the more popular they are.

## Supporting information

S1 File (DOC)

## References

[1] Haselwandter K, Winkelmann G. Ferricrocin—an ectomycorrhizal siderophore of *Cenococcum geophilum*. BioMetals, 2002, 15: 73–77. 10.1023/a:1013188823076 11860025

[2] Rinaldi AC, Comadini O, Kuyper TW. (2008) Ectomycorrhizal fungal diversity: separating the wheat from the chaff. Fungal Divers 33:1–45.

[3] Brundrett MC (2009) Mycorrhizal associations and other means of nutrition of vascular plants: understnding global diversity of host plants by resolving conflicting information and developing reliable means of diagnosis. Plant Soil 320:37–77.

[4] Tedersoo L, May TW, Smith ME. Ectomycorrhizal lifestyle in fungi: global diversity, distribution, and evolution of phylogenetic lineages. Mycorrhiza, 2010, 20: 217–263. 10.1007/s00572-009-0274-x 20191371

[5] Haselwandter H, Bowen GD. 1996 Mycorrhizal relations in trees for agroforestrty and land rehabilitation. Forest Ecol Manage, 81, 1–17.

[6] Smith SE, Read DJ. 1997 Mycorrhizal Symbiosis. San Diego: Academic Press.

[7] Wu B, Watanabe I, Hayatsu M, Nioh I. Effect of ectomycorrhizae on the growth and uptake and transport of N-15-labeled compounds by *Pinus tabulaeformis* seedlings under water-stressed conditions. *Biology and Fertility of Soil*, 1999, 28: 136–138.

[8] Bae KS, Barton LL. Alkaline phosphatase and other hydrolyases produced by *Cenococcum graniforme*, an ectomycorrhizal fungus. Applied and Environmental Microbiology, 1989, 55(10): 2511–2516. 10.1128/AEM.55.10.2511-2516.1989 16348028 PMC203113

[9] Haugen P, Simon DM, Bhattacharya D. The natural history of group I introns. TRENDS in Genetics, 2005, 21(2): 111–119. 10.1016/j.tig.2004.12.007 15661357

[10] Cech TR. Self-splicing of group I introns. Annu Rev Biochem, 1990, 59:543–568. 10.1146/annurev.bi.59.070190.002551 2197983

[11] Shinohara ML, LoBuglio KF, Rogers SO. Group-I intron family in the nuclear ribosomal RNA small subunit genes of *Cenococcum geophilum* isolates. Current Genetics, 1996, 29(4): 377–387. 10.1007/BF02208619 8598059

[12] Burke JM, Belfort M, Cech TR, Davies RW, Schweyen RJ, Shub DA, et al. Structural convention for group I introns. Nucleic Acids Research, 1987, 15(18):7217–7221. 10.1093/nar/15.18.7217 3658691 PMC306243

[13] Cech TR. Conserved sequences and structures of group-I introns: building an active site for RNA catalysis-a review. *Gene*, 1988, 73(2): 259–271. 10.1016/0378-1119(88)90492-1 3072259

[14] Michel F, Westhof E. Modeling of the three-dimensional architecture of group-I catalytic introns based on comparative sequence analysis. Journal of Molecular Biology, 1990, 216(3): 585–610. 10.1016/0022-2836(90)90386-Z 2258934

[15] Feau N, Hamelin RC, Bernier L. Variability of nuclear SSU-rDNA group I introns within *Septoria* species: incongruence with host sequence phylogenies. Journal of Molecular Evolution, 2007, 64(5): 489–499. 10.1007/s00239-005-0309-7 17457635

[16] Suh SO, Jones KG, Blackwell M (1999) A group I intron in the nuclear small subunit rRNA gene of *Cryptendoxyla hypophtoia*, an ascomycetous fungus: evidence for a new major class of group I introns. J Mol Evol 48:493–500. 10.1007/pl00006493 10198116

[17] Rogers S O, Bendich A J. Extraction of total cellular DNA from plants, algae and fungi. In: Gelvin SB, Schilperoort RA (eds) Plant Molecular Biology Manual, 2nd edn. Kluwer Academic Press, Dordrecht, The Netherlands, pp D1:1–8. 1994.

[18] Mavridou A, Cannone J, Typas M A. Identification of Group-I Introns at Three Diff- erent positions within the 28S rDNA Gene of the entomopathogenic fungus *Metarhizium anisopliae* var. Fungal Genet Biology, 2000, 31(2):79–90. 10.1006/fgbi.2000.1232 11170737

[19] White TJ, Bruns T, Lee S, Taylor J, 1990. Amplification and direct sequencing of fungal ribosomal RNA genes for phylogenetics. In: Innis MA, Gelfand D, Sninsky J, White T. PCR Protocols. A guide to methods and applications. New York, USA: Academic Press, 315–322.

[20] Cannone JJ, Subramanian S, Schnare MN. The Comparative RNA Web (CRW) Site: an online database of comparative sequence and structure information for ribosomal, intron, and other RNAs. BMC Bioinformatics 2002, 3:2. 10.1186/1471-2105-3-2 11869452 PMC65690

[21] Zuker M. Mfold web server for nucleic acid folding and hybridization prediction. Nucleic Acids Research, 2003, 31: 3406–3415. 10.1093/nar/gkg595 12824337 PMC169194

[22] Mathews D.H., Disney M. D., Childs J. L., Schroeder S. J., Zuker M. and Turner D.H. (2004) Incorporating chemical modification constraints into a dynamic programming algorithm for prediction of RNA secondary structure. Proc. Natl. Acad. Sci. USA 101:7287–7292. 10.1073/pnas.0401799101 15123812 PMC409911

[23] Wikmark OG, Einvik C, De Jonckheere JF, Johansen SD. Short-term sequence evolution and vertical inheritance of the *Naegeria* twin-ribozyme group I intron. BMC Evolutionary Biology, 2006, 6:39. 10.1186/1471-2148-6-39 16670006 PMC1464144

[24] Nielsen H, Johansen S. Group I introns. RNA Biology, 2009, 6(4):375–383. 10.4161/rna.6.4.9334 19667762

[25] Oliveira MC, Ragan MA. Variant forms of a group-I intron in nuclear small-subunit rRNA genes of the marine red alga *Porphyra spiralis* var. amplifolia. Molecular Biology and Evolution, 1994, 11(2): 159–207. 10.1093/oxfordjournals.molbev.a040100 8170361

[26] Nikoh N, Fukatsu T. Evolutionary dynamics of multiple group I introns in nuclear ribosomal RNA genes of endoparasitic fungi of the genus *Cordyceps*. Molecular Biology and Evolution, 2001, 18(9): 1631–1642. 10.1093/oxfordjournals.molbev.a003952 11504844

[27] Perotto S, Nepote-Fus P, Saletta L, Bandi C, Young JPW. A diverse population of introns in the nuclear ribosomal genes of ericoid mycorrhizal fungi includes elements with sequence similarity to endonuclease-coding genes. Molecular Biology and Evolution, 2000, 17(1): 44–59. 10.1093/oxfordjournals.molbev.a026237 10666705

[28] Hoshina R, Kamako SI, Imamura N. Three Group-I introns in 18S rDNA of Endosymbiotic Algae of *Paramecium bursaria* from Japan. American Institute of Physics, 2004, 203–205.

[29] Hoshina R, Imamura N. Eu-Chlorella large subunit rDNA sequences and group I introns in ribosomal DNA of the paramecian symbiotic alga NC64A. Phycological Research, 2008; 56: 21–32.

[30] Wang T, Chen LH. Group-I introns in 18S rDNA of *Cenococcum geophilum* Fr. Acta Microbiologica Sinica, 2012, 52(9): 1059–1067 (in Chinese) 23236839

[31] Engelhardt MA, Doherty EA, Knitt DS, Doudna JA, Herschlag D. The P5abc peripheral element facilitates preorganization of the tetrahymena group I ribozyme for catalysis. Biochemistry, 2000, 39(10): 2639–2651. 10.1021/bi992313g 10704214

[32] Haugen P, Reeb V, Lutzoni F, Bhattacharya D. The evolution of homing endonuclease genes and group I introns in nuclear rDNA. Molecular Biology and Evolution, 2004, 21(1): 129–140. 10.1093/molbev/msh005 14595099

[33] Chevalier BS and Stoddard SL. Homing endonucleases: structural and functional insight into the catalysts of intron/intein mbility. Nucleic Acids Research, 2001, 29(18): 3757–3774. 10.1093/nar/29.18.3757 11557808 PMC55915

[34] Flick KE, Jurica MS, Monnat RJ Jr and Stoddard BL. DNA binding and cleavage by the nuclear intron-encoded homing endonuclease I-PpoI. Nature, 1998, 394: 96–101. 10.1038/27952 9665136

[35] Elde M, Haugen P, Willassen NP, Johansen S. I-NjaI, a nuclear intron–encoded homing endonuclease from *Naegleria*, generates a pentanucleotide 3’ cleavage-overhang within a 19 base-pair partially symmetric DNA recognition site. Eur. J. Biochem., 1999, 259: 281–288. 10.1046/j.1432-1327.1999.00035.x 9914504

[36] Johansen S, Einvik C, Nielsen H. DiGIR1 and NaGIR1: naturally occurring group I-like ribozymes with unique core organization and evolved biological role. Biochimie, 2002, 84: 905–912. 10.1016/s0300-9084(02)01443-8 12458083

[37] Birgisdottir AB and Johansen S. Site-specific reverse splicing of a HEG-containing group I intron in ribosomal RNA. Nucleic Acids Research, 2005, 33(6): 2042–2051. 10.1093/nar/gki341 15817568 PMC1074745

[38] Belfort M. and Roberts R.J. (1997) Homing endonucleases: keeping the house in order. Nucleic Acids Res., 25, 3379–3388. 10.1093/nar/25.17.3379 9254693 PMC146926

[39] Decatur WA, Johansen S, Vogt VM. Expression of the *Naegleria* intron endonuclease is dependent on a functional group I self-cleaving ribozyme. RNA, 2000, 6: 616–627. 10.1017/s1355838200992203 10786852 PMC1369942

[40] Gogarten JP and Hilario E. Inteins, introns, and homing endonucleases: recent revelations about the life cycle of parasitic genetic elements. BMC Evolutionary Biology, 2006, 6:94. 10.1186/1471-2148-6-94 17101053 PMC1654191

[41] Douhan GW, Rizzo DM. Phylogenetic divergence in a local population of the ectomycorrhizal fungus *Cenococcum geophilum*. New Phytologist, 2005, 166(1): 263–271. 10.1111/j.1469-8137.2004.01305.x 15760369

[42] Goddard MR and Burt A. Recurrent invasion and extinction of a selfish gene. Proc. Natl Acad. Sci. USA, 1999, 96(24): 13880–13885. 10.1073/pnas.96.24.13880 10570167 PMC24159

[43] Weeks KM, Cech TR. Protein Facilitation of Group I Intron Splicing by Assembly of the Catalytic Core and the 5’ Splice Site Domain. Cell, 1995, 82: 221–230. 10.1016/0092-8674(95)90309-7 7628013

[44] Kruger K., Grabowski P.J., Zaug A.J., Sands J., Gottschling D.E., and Cech T.R. (1982). Self-splicing RNA: autoexcision and autocyclization of the ribosomal RNA intervening sequence of *Tetrahymena*. Cell 31, 147–157. 10.1016/0092-8674(82)90414-7 6297745

[45] Guo F, Gooding AR, Cech TR. Structure of the *Tetrahymena* ribozyme: base triple sandwich and metal ion at the active site. Molecular Cell, 2004, 16: 351–362. 10.1016/j.molcel.2004.10.003 15525509

[46] Adams PL, Stahley MR, Gill ML, Kosek AB, Wang JM, Strobel SA. Crystal structure of a group I intron splicing intermediate. RNA, 2004, 10: 1867–1887. 10.1261/rna.7140504 15547134 PMC1370676

[47] Horst GV, Christlan A, Inoue T. Reconstitution of a group I intron self-splicing reaction with an activator RNA. Proc. Nail. Acad. Sci. USA, 1991, 88: 184–188. 10.1073/pnas.88.1.184 PMC50774 1986364

[48] Guo QB, Lambowitz AM. A tyrosyl-tRNA synthetase binds specifically to the group I mtron catalytic core. Genes Dev. 1992, 6: 1357–1372. 10.1101/gad.6.8.1357 1379562

[49] Doudna JA, Cormack BP, Szostak JW. RNA structure, not sequence, determines the 5’ splice-site specificity of a group I intron. Proc. Natl. Acad. Sci. USA, 1989, 86: 7402–7406. 10.1073/pnas.86.19.7402 2678103 PMC298070

