# Supplementary material for: Analysis of small and large subunit rDNA introns from several ectomycorrhizal fungi species
Source: PLoS One. 2021 Mar 15;16(3):e0245714. doi: 10.1371/journal.pone.0245714 (PMC7959364; doi:10.1371/journal.pone.0245714)
Supplement: S1 File — (DOC) [file pone.0245714.s001.doc]

**18S rDNA introns:**

YUN KF424613

4-1 KF424605

3-1 KF424603

Spop3 KF424608

Spicea JX281795

Yang1 JX093580

CG417 JX093575

CG JX093576

SB1 JX093578

Spop1 JX093579

CG5 JX093573

CG54 JX093574

O5 JX093582

1-1 MW567472

Picea-I1 MW644975

Picea-I2 MW644976

Pop7 MW644977

Baihua MW644978

Shanbai MW644979

Yang2 MW644980

AM51 MW644981

SB5 MW644982

SB6 MW644983

**28S rDNA introns:**

O1-L MW644984

2-3-L MW644985

2-8-L MW644986

3-1-L MW644987

SB5-L MW644988

WL-L MW644989

Spicea-L MW644990

2010Cg-L MW644991

CG5-L MW644992

CG54-L MW644993

AM51-IL1 MW644994

AM51-IL2 MW644995

Yang2-IL2 MW644996

Shanbai-L MW644997

Baihua-L MW644998

Pop7-L MW644999

Pop2-L MW645000

2-15-L MW645001

SO5-L MW645002

2-16 MW654482
