# Supplementary material for: Analysis of small and large subunit rDNA introns from several ectomycorrhizal fungi species
Source: PLoS One. 2021 Mar 15;16(3):e0245714. doi: 10.1371/journal.pone.0245714 (PMC7959364; doi:10.1371/journal.pone.0245714)
Supplement: Attachment — Submitted filename: Response to Reviewers letter-r.docx [file pone.0245714.s002.docx]

**Rebuttal letter**

**Journal Requirements:**

1. Please ensure that your manuscript meets PLOS ONE's style requirements, including those for file naming.

I have downloaded the PLOS ONE style templates, and revised the manuscript according to the templates.

2. In your Methods section, please provide additional location information, including geographic coordinates for the data set if available.

Location information for sampling has been added in the Methods section. "Tested strains were isolated from sclerotial bodies as well as mycorrhizae samples which were collected from Daqing Mountain (longitude 111.25°-112.30°, Latitude 40.35°-40.57°) with permission from Inner Mongolia Daqing Mountain Nature Reserve, Helan Mountain (longitude 105.40°-105.58°, Latitude 38.10°-39.08°) with permission from Helan Mountain National Nature Reserve, Daxingan Mountainn (longitude 121.30°-121.31°, Latitude 50.49°-50.51°) with permission from Genhe ecological positioning station in Daxingan Mountainn of Inner Mongolia, and Wula Mountain (longitude 108.2°-108.5°, Latitude 40.9°- 40.41°) with permission from Inner Mongolia Wula Mountain National Forest Park in Inner Mongolia of China. No specific permits were required as the research did not include the destruction of vegetation."

3. In your Data Availability statement, you have not specified where the minimal data set underlying the results described in your manuscript can be found. PLOS defines a study's minimal data set as the underlying data used to reach the conclusions drawn in the manuscript and any additional data required to replicate the reported study findings in their entirety. All PLOS journals require that the minimal data set be made fully available.

In abstract section, “We found that conserved 1, 2, 3 regions in introns 2-16-I and SO5-I with HEG are less conservative than as the introns without HEG did.” has been deleted.

4. Competing Interests:

The coauthors (Wei Yan, Ting Wang, Lian Liu, and Zhuo Yu) have declared that no competing interests exist. The coauthor (Yu Wang) was my former graduate student who had taken part in this work, he did not return my email. I did not know how to revise the submission competing interests form and delete“We thank professor Zhiwu Li from School of Mechano-Electronic Engineering of XiDian University of China for assistance in data analysis”.

5. Please amend the manuscript submission data (via Edit Submission) to include authors Mark Gomelsky and Larry R Peterson.

Coauthors Mark Gomelsky and Larry R Peterson have been deleted. DNA sequences will be submitted to GenBank, but they are not released to the public database until this article is published.

6. PLOS requires an ORCID iD for the corresponding author in Editorial Manager on papers submitted after December 6th, 2016. Please ensure that you have an ORCID iD and that it is validated in Editorial Manager.

My ORCID iD is [https://orcid.org/0000-0001-7829-7076](https://orcid.org/0000-0001-7829-7076).

**Additional Editor Comments:**

The reviewer gave very good suggestions of how to improve the submission. In general, it must be made clear that the analyzed strains were pure, were what was identified when looking at the rhizomorphs, and also to compare to genomic data to make sure that different rRNA genes present in one strain (multiple rRNA genes in one genome as well as two nuclei in the respective dikaryon that was isolated) are not mixed.

Identification of the isolated fungi were to compare 18S rDNA and ITS regions sequences to GenBank data. Sequencing figures were clear and not multiple peak, we think strains were pure.

**Review Comments to the Author:**

1. Agarose gel images of the PCR amplicons could be a good additive in the text. May be include as supplementary figs. The gel images would give a quick view on the presence and absence of introns based on the size variations. What were the size of the amplicons? How clean was the PCR amplification?

I agree with agarose gel images of The PCR amplicons as supplementary figs.

2. The authors indicated that ‘Pure cultures of tested strains were isolated from sclerotial bodies as well as mycorrhizae samples which were collected….’ How did they ensure ‘purity’ in the metacommunity at the time of sampling? If these are already existing stock cultures, please provide ATCC or MTCC accession numbers in the table. This is important to know as any minute ‘noise/impurity’ would be amplified through PCR/sequencing, which would further affect the downstream analysis.

In Fungal strains and DNA extraction of material and method section, Pure cultures of tested strains refer to the cultured colony mycelium, were not tested directly mycorrhizae or sclerotial bodies. Identification of the isolated fungi were to compare 18S rDNA and ITS regions sequences to GenBank data. Sequencing figures were clear and not multiple peak, we think strains were pure. “Pure culture of” were deleted.

3. While revising your submission, please upload your figure files to the Preflight Analysis and Conversion Engine (PACE) digital diagnostic tool. PACE helps ensure that figures meet PLOS requirements.

I uploaded the manuscript figures to the Preflight Analysis and Conversion Engine (PACE) digital diagnostic tool, the figures meet PLOS requirements. Fig.3 and Fig.4 have been revised and made more clear.

4. If applicable, we recommend that you deposit your laboratory protocols in protocols.io to enhance the reproducibility of your results. Protocols.io assigns your protocol its own identifier (DOI) so that it can be cited independently in the future.

I have tried to deposit my laboratory protocols in protocols.io, but I did not know to use protocols.io. after seeing the instructions ([http://journals.plos.org/plosone/s/submission-guidelines#loc-laboratory -protocols](http://journals.plos.org/plosone/s/submission-guidelines#loc-laboratory-protocols)).
